# Supplementary material for: Structural insights into the selective recognition of RF-amide peptides by neuropeptide FF receptor 2
Source: EMBO Rep. 2025 Mar 24;26(9):2413–34. doi: 10.1038/s44319-025-00428-2 (PMC12069643; doi:10.1038/s44319-025-00428-2)
Supplement: Supplementary file 2 — Appendix [file 44319_2025_428_MOESM2_ESM.pdf]

Structural insights into the selective recognition of RF-amide peptides by neuropeptide FF receptor 2

Jeesoo Kim, Sooyoung Hong, Hajin Lee, Hyun Sik Lee, Chaehee Park, Jinuk Kim, Wonpil Im, Hee-Jung Choi

Appendix Figure S1 2
Appendix Figure S2 3
Appendix Figure S3 4
Appendix Figure S4 5
Appendix Figure S5 6
Appendix Figure S6 7
Appendix Figure S7 8
Appendix Figure S8 9
Appendix Figure S9 10
Appendix Figure S10 11
Appendix Figure S11 12
Appendix Figure S12 13
Appendix Figure S13 14
Appendix Table S1 15
Appendix Table S2 16
References 17

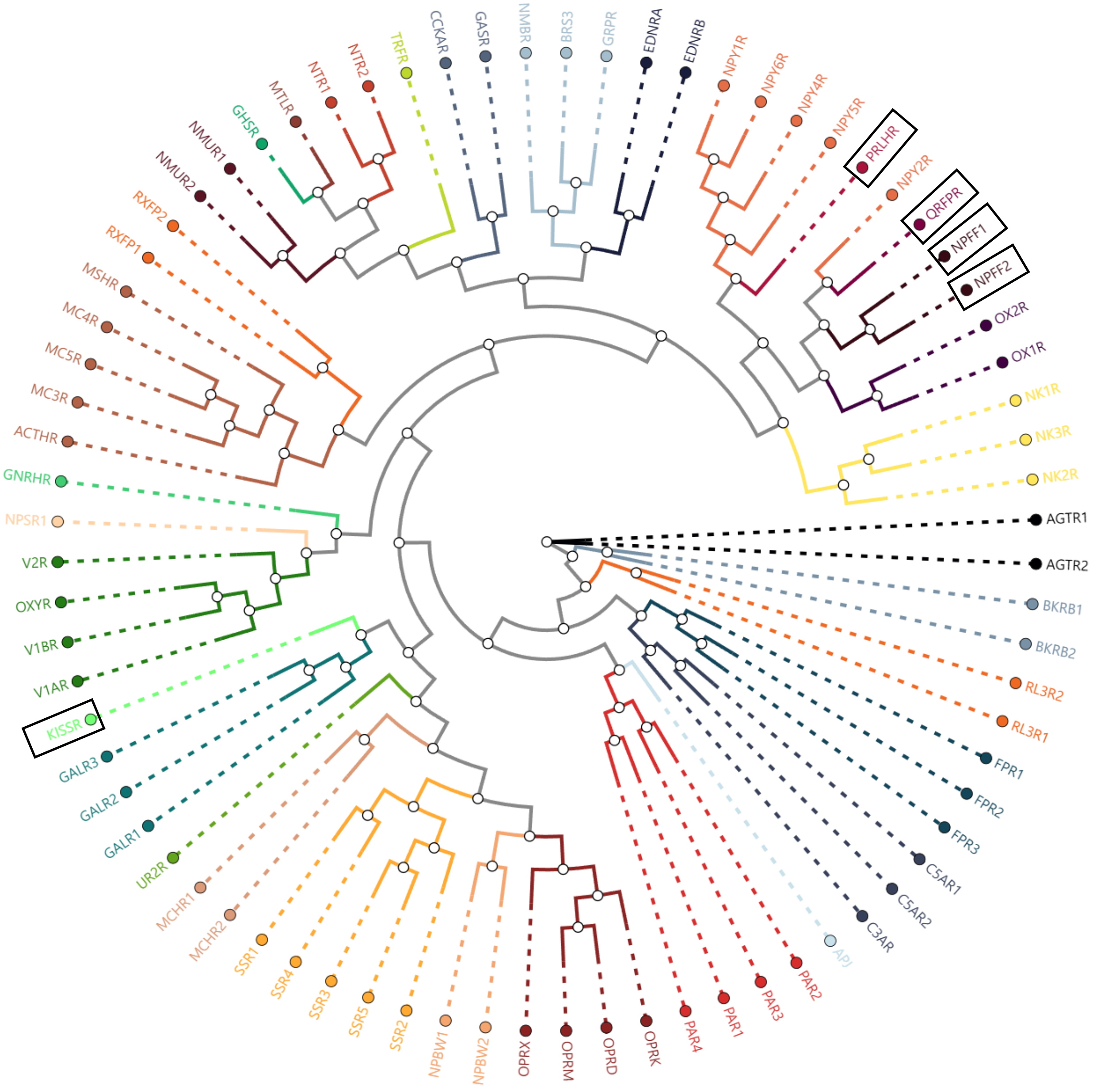

Appendix Figure S1. Phylogenetic tree of Class A peptide GPCRs
Phylogenetic tree of Class A peptide GPCRs generated using GPCRdb[1]. The RF-amide receptors, including NPFFR2 (NPFF2), NPFFR1 (NPFF1), QRFPR, PrRPR (PRHLR), and KISS1R (KISSR), are marked with black boxes.

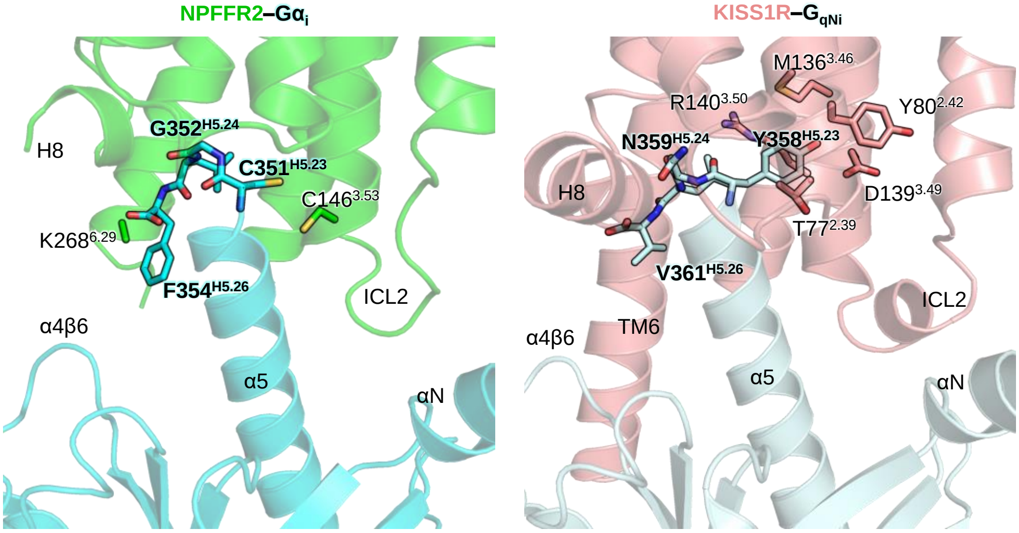

Appendix Figure S2. Comparison of the G protein binding regions of NPFFR2 and KISS1R

Superposition of the hNPSF–NPFFR2–G_i complex and the Kisspeptin-10–KISS1R–G_{qNi} (PDB: 8ZJD) based on the receptors reveals that NPFFR2 and KISS1R show different interaction pattern toward H5.23 residue of Gα. NPFFR2 and KISS1R follow the color code in **Fig. 3**, and Gα proteins bound to each receptor are colored cyan and light cyan, respectively.

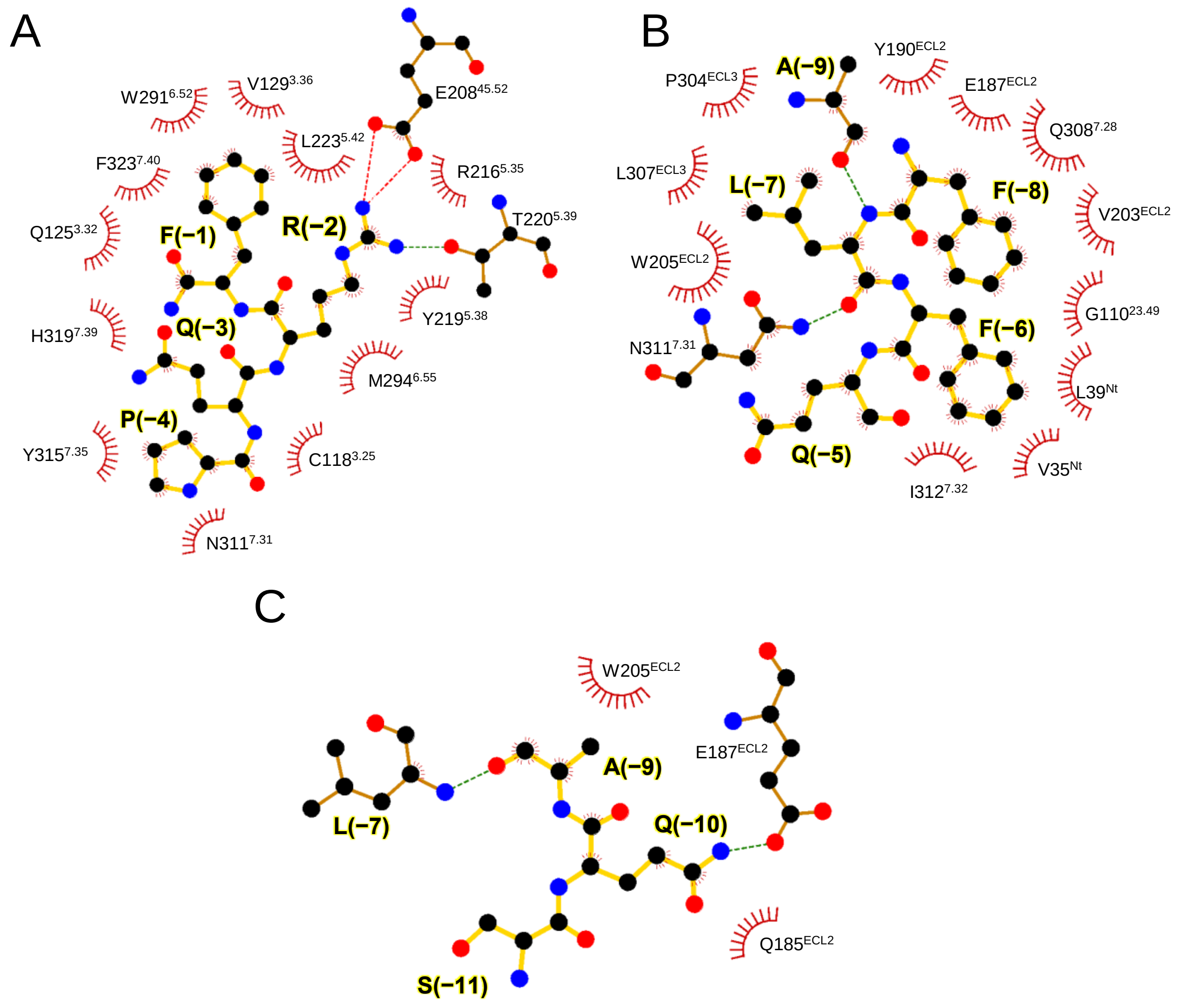

Appendix Figure S3. Schematic representations of hNPSF–NPFFR2 interaction using LigPlot+
The interactions between hNPSF and NPFFR2 are analyzed using the LigPlot+[2] program. The hydrogen bonds and the salt bridges are shown in green and red dashed lines, respectively. **A**, **B**, and **C** show detailed interactions shown in red, blue, and yellow boxes in **Fig. 2.**

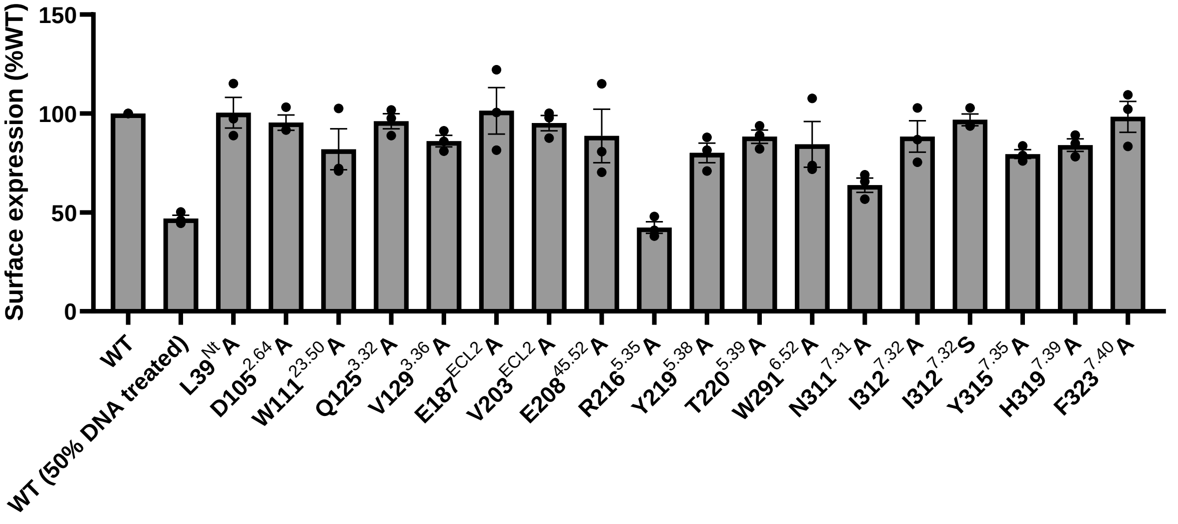

Appendix Figure S4. ELISA-based surface expression assay of NPFFR2 mutants

The surface expression levels of NPFFR2 mutants were analyzed with an ELISA-based surface expression assay prior to forskolin-stimulated cAMP assays. The bars and error bars for each sample represent the mean and standard error of the mean (SEM.) of three independent experiments, respectively. Each circle represents an individual data point from the experiment. The surface expression values normalized to WT are summarized in Table EV1.

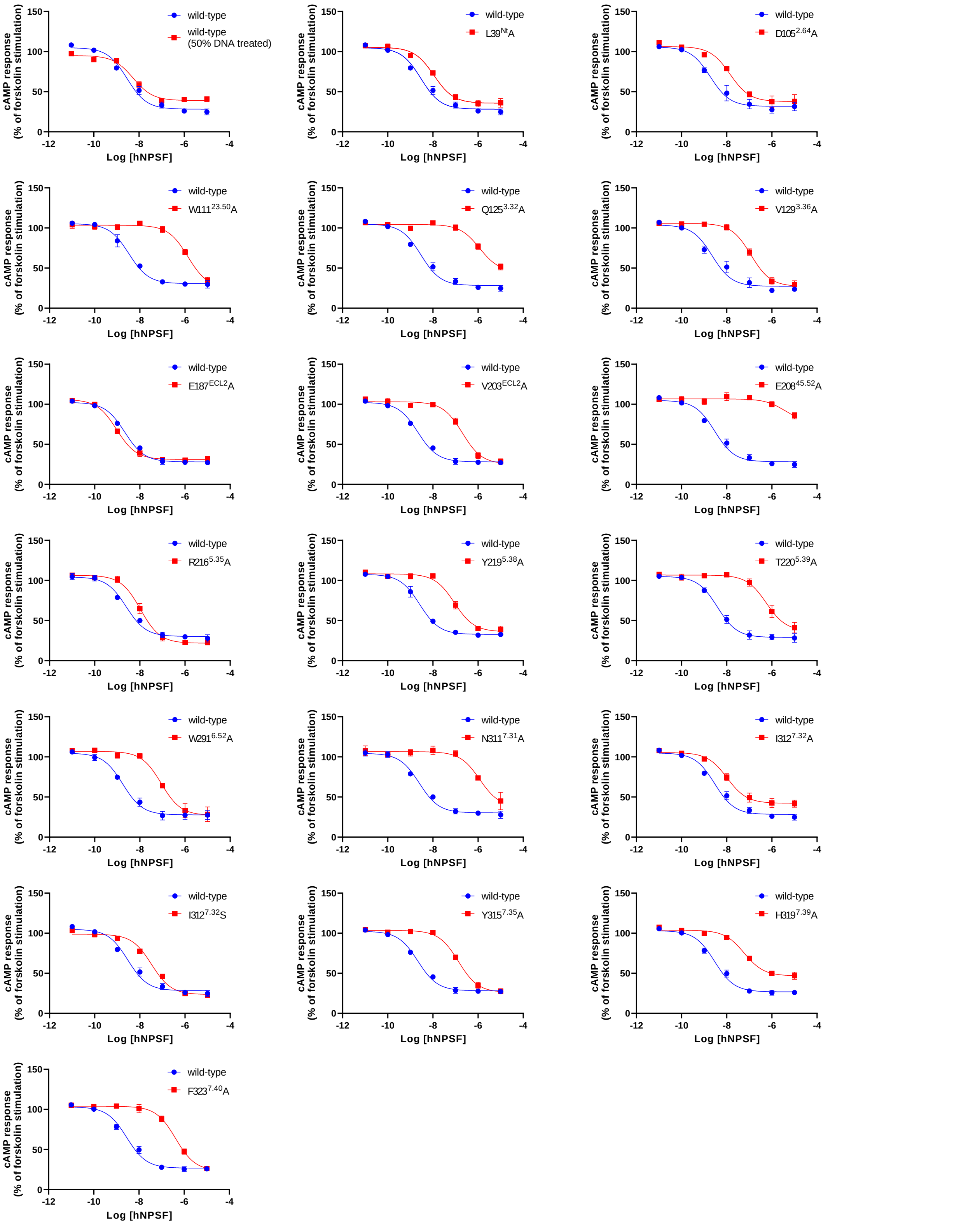

Appendix Figure S5. GloSensor^{TM} cAMP assays of mutants and wild-type NPFFR2.
The dose-response of cAMP assay data for each mutant (red) presented in **Fig. 2B** are compared against the wild-type NPFFR2 (blue). Each experiment was conducted after adjusting the mutant's expression level to that of the wild-type NPFFR2 (**Appendix Fig. S3**), and pcDNA3.1 was used to match the total amount of DNA transfected. Data points represent mean values from three independent experiments (each with technical triplicate), with error bars indicating SEM. Raw data were normalized to the vehicle-treated (0%) and 0.5 μM forskolin-treated (100%) responses. The Emax, EC_{50}, error, and p-value values are summarized in Table EV1.

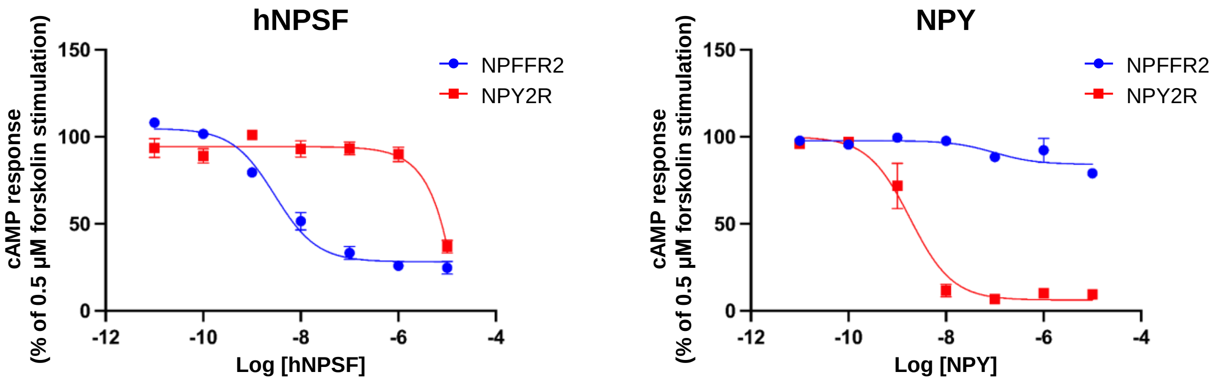

Appendix Figure S6. cAMP response to hNPSF and NPY on NPFFR2 and NPY2R.
The cAMP response to the ligands hNPSF and NPY was measured for NPFFR2 and NPY2R receptors using a forskolin-stimulated cAMP assay. The symbols represent mean values from three independent experiments, each conducted in technical triplicates, with error bars indicating SEM. Responses were normalized to vehicle-treated controls (0%) and 0.5 µM forskolin-treated controls (100%).

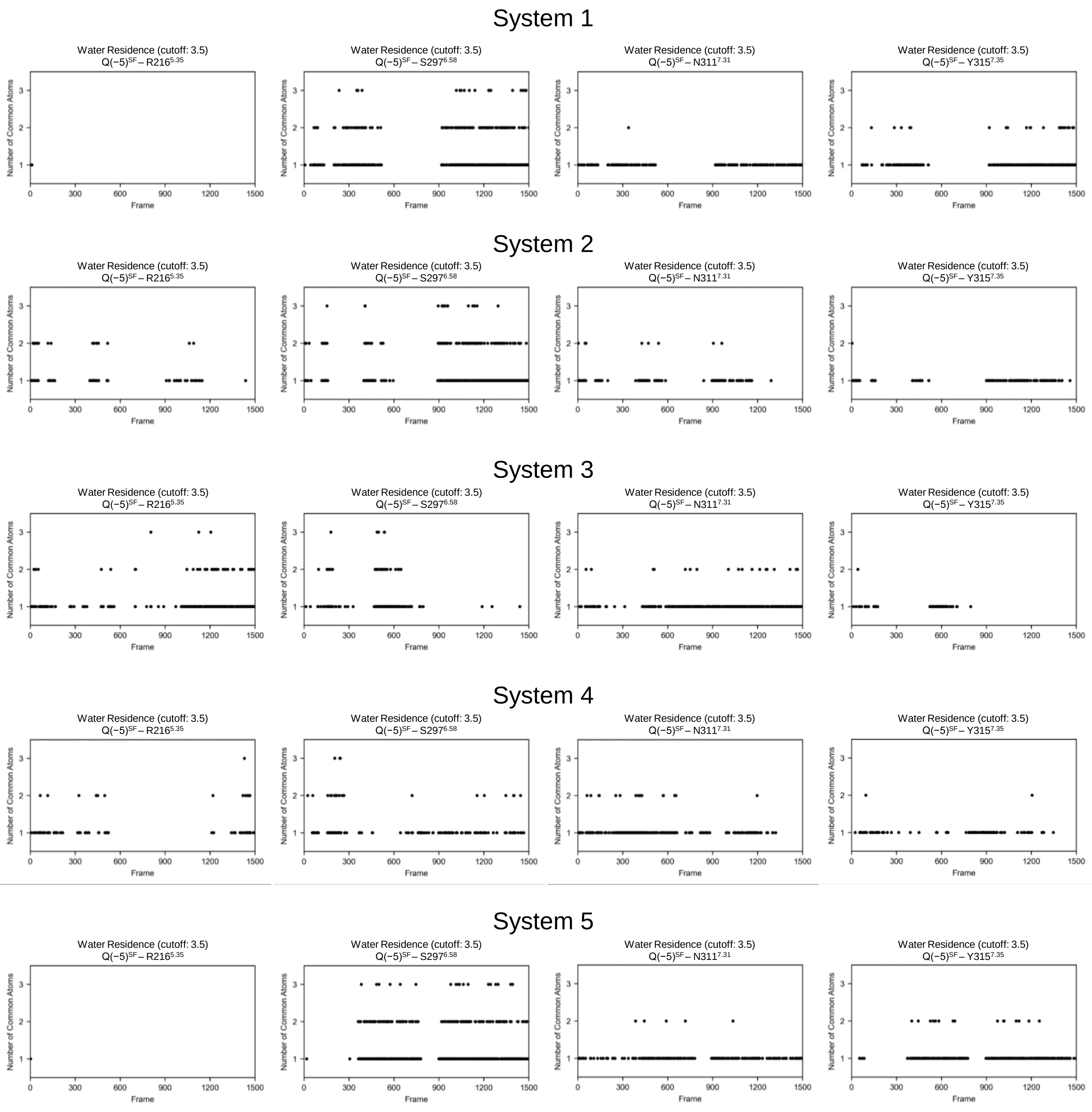

Appendix Figure S7. Water-mediated interactions between hNPSF Q(−5) and NPFFR2
We investigated the number of water molecules within a hydrogen-bond distance (3.5 Å) between Q(−5) of hNPSF and NPFFR2 residues (R216$^{5.25}$, S297$^{6.58}$, N311$^{7.31}$, Y315$^{7.35}$) throughout the five different systems of 1.5 μs-long MD simulations.

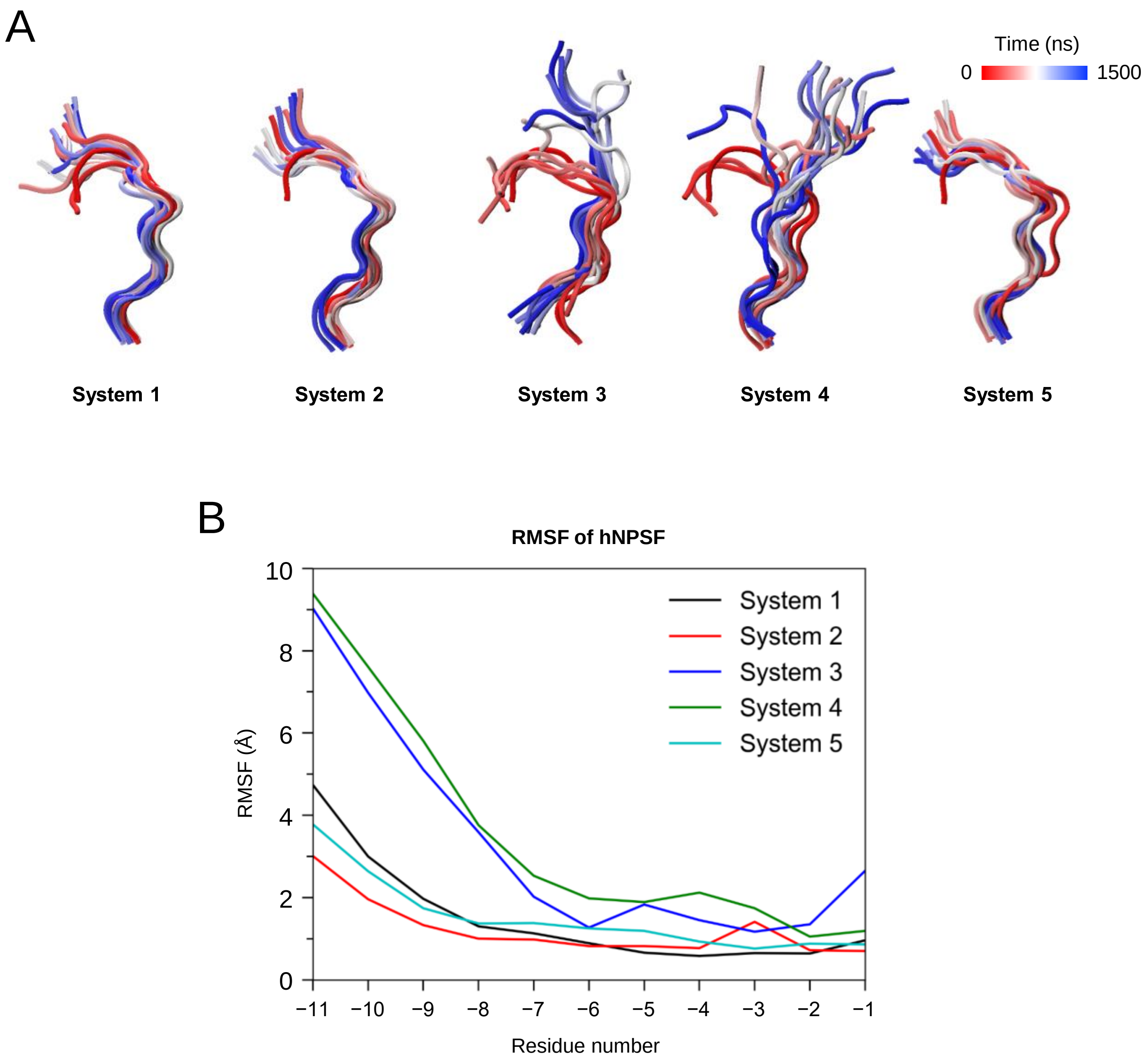

Appendix Figure S8. Fluctuation analysis of hNPSF using MD simulation.
(A) Structural alignment of hNPSF models from 5 different systems throughout 1.5 μs-long MD simulations, segmented by 100 ns intervals. **(B)** RMSF (root-mean-square fluctuation) values of hNPSF are calculated across the five systems and plotted against residue numbers to show the fluctuation profile of each residue.

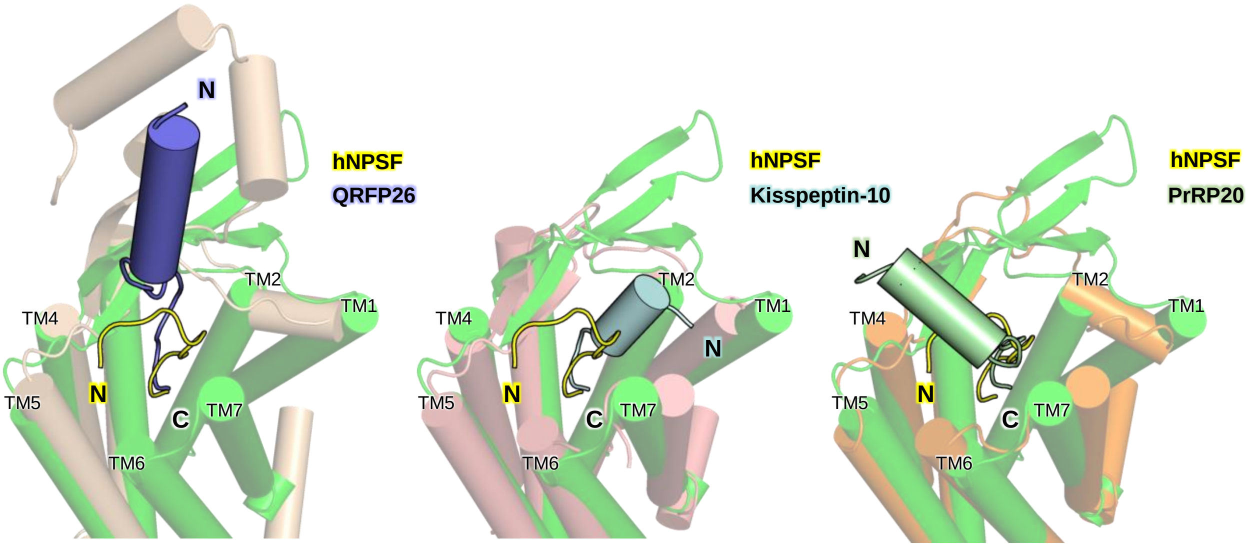

Appendix Figure S9. Structural alignment of RF-amide receptors and comparison of their ligand binding modes

Cartoon models of hNPSF-bound NPFFR2, QRFP26-bound QRFPR, Kisspeptin-10-bound KISS1R, and PrRP20-bound PrRPR are shown with cylindrical helices. The N and C-termini of each peptide ligand show that hNPSF, QRFP26, Kisspeptin-10, and PrRP20 share a similar binding pose at the C-terminus, while their N-terminal interactions differ significantly. Each protein is colored consistently with the color scheme used in Figure 3.

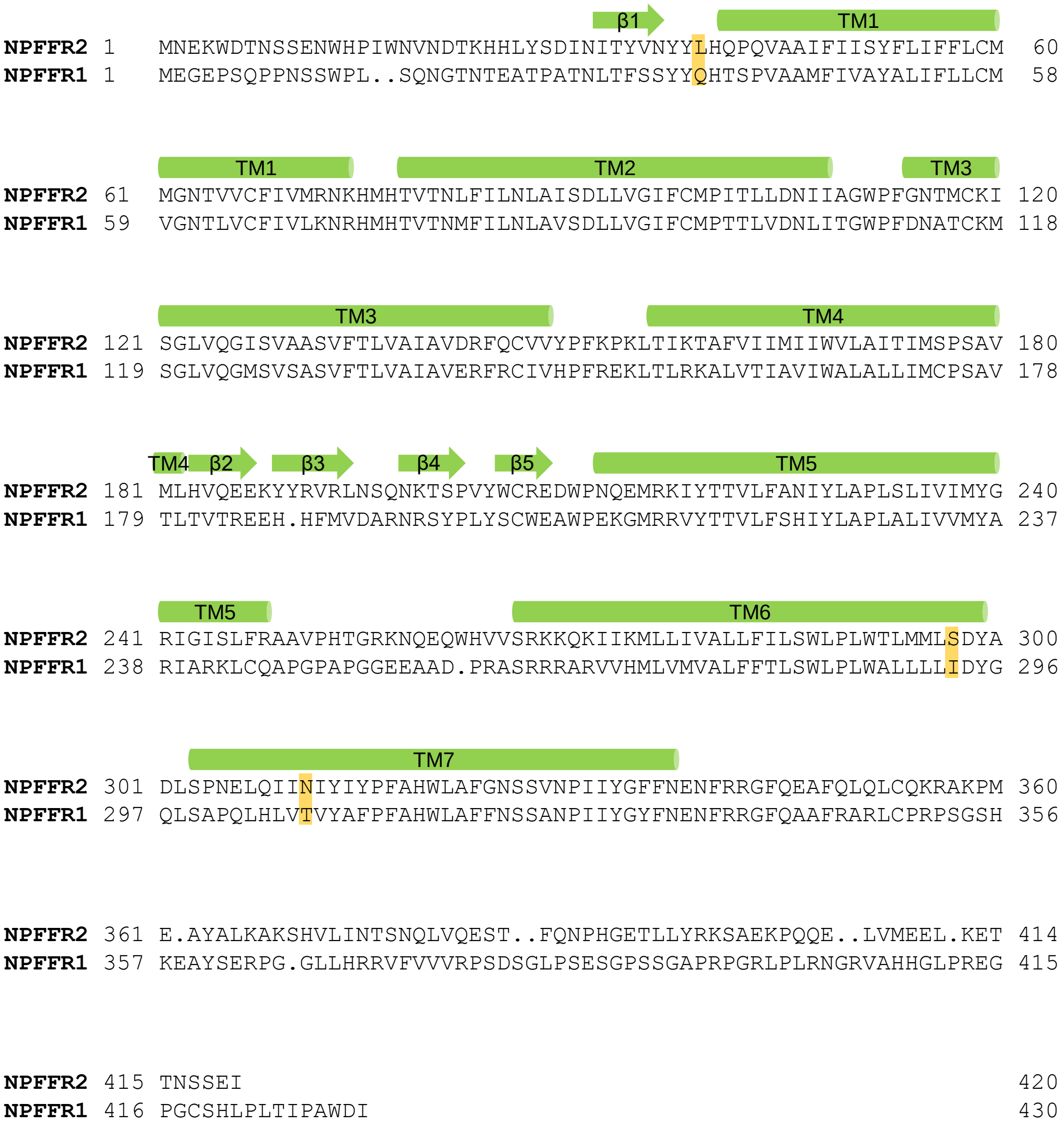

Appendix Figure S10. Sequence alignment between NPFFR2 and NPFFR1

Sequence alignment between human NPFFR2 and human NPFFR1 is shown with the secondary structure of NPFFR2 on top. Three residues mutated in the NPFFR1 chimera (Q37^{Nt}L/I293$^{6.58}$S/T307$^{7.31}$N), are highlighted in yellow.

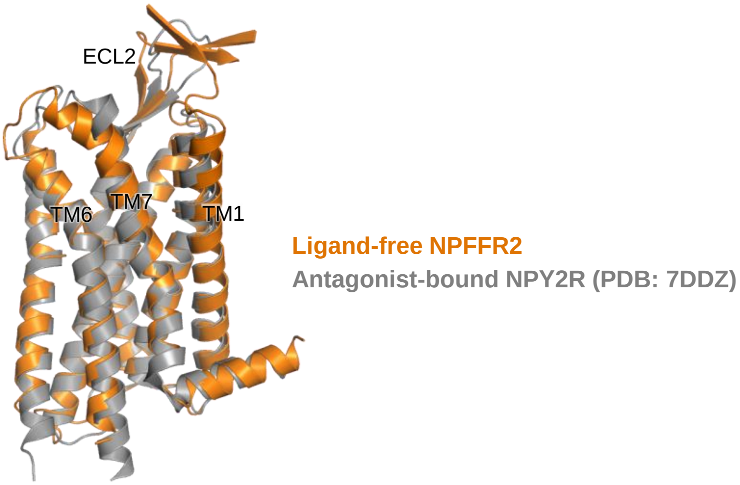

Appendix Figure S11. Structural alignment of ligand-free NPFFR2 and inactive NPY2R
The TMD of ligand-free NPFFR2 from the NPFFR2-BRIL–Fab^{BRIL}–Nb complex structure and antagonist-bound NPY2R structure (PDB: 7DDZ) are aligned. The structure of ligand-free NPFFR2 closely resembles NPY2R in its inactive state with an RMSD value of 1.0 Å.

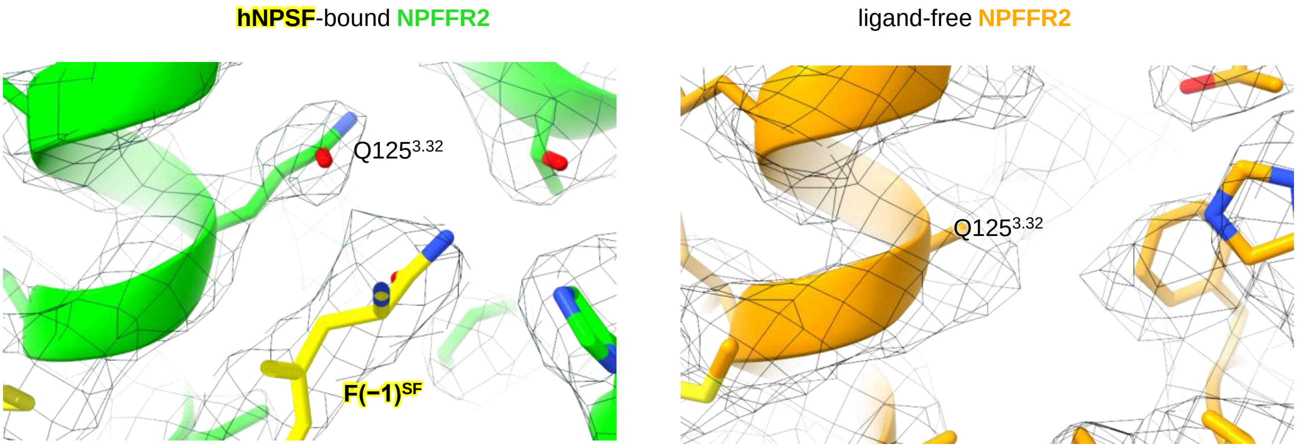

Appendix Figure S12. The electron density map of Q125$^{3.32}$ observed in the active structure and ligand-free state structure of NPFFR2.

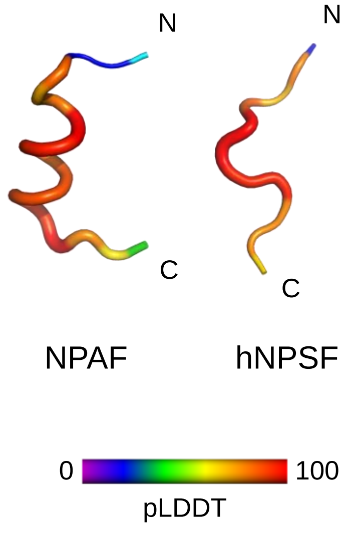

Appendix Figure S13. Alphafold3-predicted model of RF-amide peptide NPAF
The structures of NPAF and hNPSF were predicted using AlphaFold3[3] with limited input of amino acid sequences and without C-terminal amidation. Colors represent the pLDDT score, ranging from 0 to 100.

Peptide Name	Sequence	Receptor
GnIH (RFRP-1)<br>NPVF (RFRP-3)	MPHSFANLPLRF-NH_2<br>VPNLPQRF-NH_2	NPFFR1
hNPSF<br>NPFF<br>NPAF	SQAFLFQPQRF-NH_2<br>FLFQPQRF-NH_2<br>AGEGLNSQFWSLAAPQRF-NH_2	NPFFR2
Kisspeptin-10	YNWNSFGLRF-NH_2	KISS1R
PrRP20	TPDINPAWYASRGIRPVGRF-NH_2	PrRPR
QRFP26	VGTALGSLAGELNGYNRKKGGFSFRF-NH_2	QRFPR

Appendix Table S1. Various RF-amide peptides and their receptors

	**hNPSF–NPFFR2–G_i–scFv16**<br>PDB code: 9JFY<br>EMD ID: EMD-61444	**NPFFR2-BRIL–Fab^{BRIL}–Nb^{Fab}**<br>PDB code: 9JG0<br>EMD ID: EMD-61446
**Data collection and processing**		
**Magnification**	75,000	81,000
**Voltage (kV)**	300	300
**Electron exposure (e^-/Å^2)**	45	60
**Defocus range (μm)**	−0.8 ~ −2.0	−0.8 ~ −2.0
**Pixel size (Å)**	1.05	1.05
**Symmetry imposed**	C1	C1
**Initial particle projections (no.)**	6,152,021	6,231,249
**Final particle projections (no.)**	310,911	324,660
**Map resolution (Å)**	3.2	2.9
**FSC threshold**	0.143	0.143
**Refinement**		
**Initial model used (PDB code)**	7YON	7TUY
**Model composition**		
Non-hydrogen atoms	8,805	7,271
Protein residues	1,133	950
Ligand	NFA: 1	N/A
Water	N/A	N/A
**R.m.s. deviations**		
Bond lengths (Å)	0.004	0.004
Bond angles (°)	0.902	0.954
**Validation**		
MolProbity score[4]	1.28	1.46
Clashscore	5.28	8.63
Rotamer outliers (%)	0.00	0.13
CaBLAM outliers (%)	1.19	0.98
**Ramachandran plot**		
Favored (%)	98.11	98.08
Allowed (%)	1.89	1.92
Disallowed (%)	0.00	0.00

Appendix Table S2. Cryo-EM data collection, model refinement and validation statistics.

References

1. Pandy-Szekeres G, Munk C, Tsonkov TM, Mordalski S, Harpsoe K, Hauser AS, Bojarski AJ, Gloriam DE (2018) GPCRdb in 2018: adding GPCR structure models and ligands. Nucleic Acids Res 46: D440-D446
2. Laskowski R. A., Swindells M. B. LigPlot+: Multiple Ligand-Protein Interaction Diagrams for Drug Discovery. *J Chem Inf Model* **51**, 2778-2786 (2011).
3. Abramson J., *et al*. Accurate structure prediction of biomolecular interactions with AlphaFold 3. *Nature* **630**, 493-500 (2024).
4. Prisant M. G., Williams C. J., Chen V. B., Richardson J. S., Richardson D. C. New tools in MolProbity validation: CaBLAM for CryoEM backbone, UnDowser to rethink "waters," and NGL Viewer to recapture online 3D graphics. *Protein Sci* **29**, 315-329 (2020).
