# Supplementary figures and images for: Structural insights into the selective recognition of RF-amide peptides by neuropeptide FF receptor 2
Source: EMBO Rep. 2025 Mar 24;26(9):2413–34. doi: 10.1038/s44319-025-00428-2 (PMC12069643; doi:10.1038/s44319-025-00428-2)

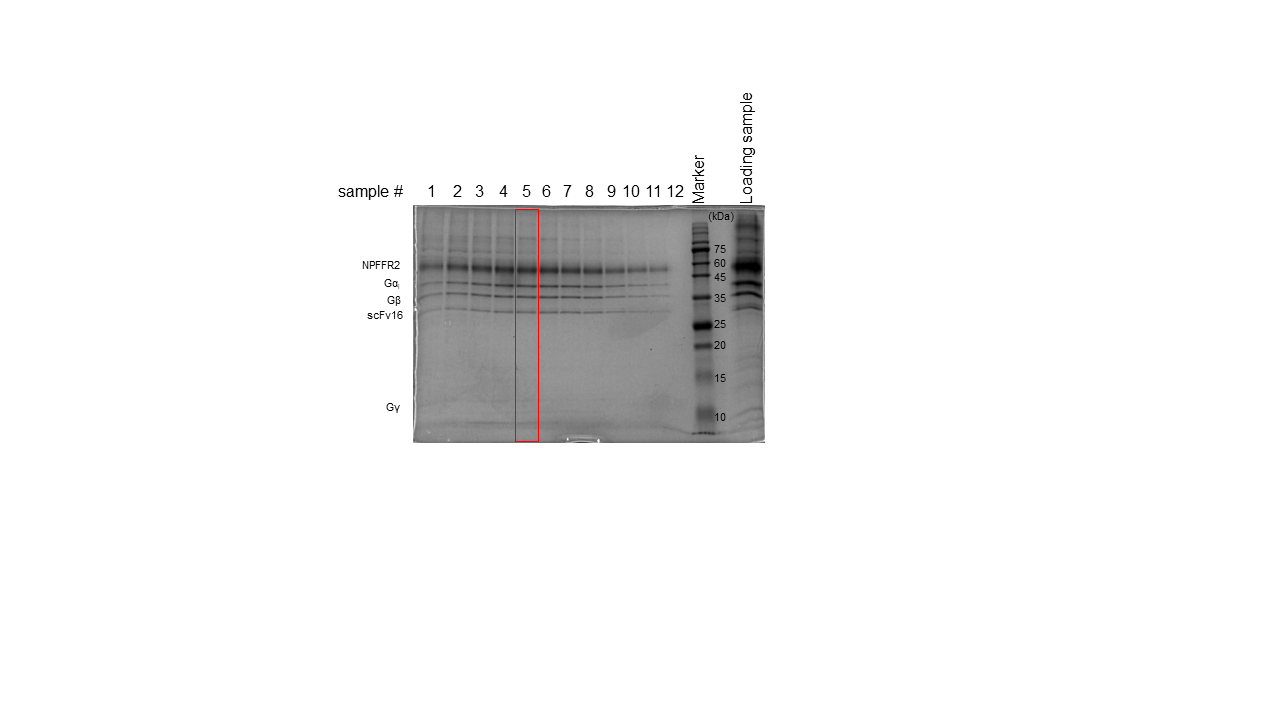

Supplement: Supplementary file 7 — Figure EV & Appendix Source Data [file 44319_2025_428_MOESM7_ESM.zip › Source Data for Expanded View and Appendix/FigureEV1/B/SDS_PAGE_raw_data.tif]

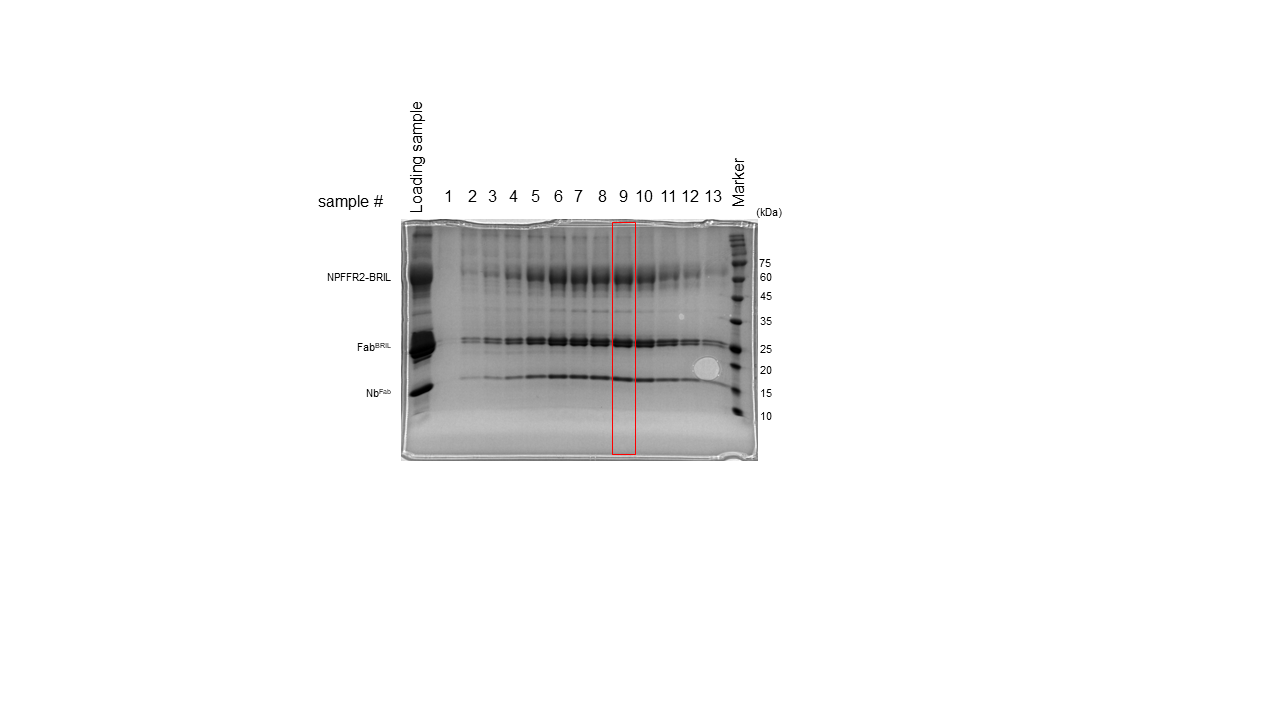

Supplement: Supplementary file 7 — Figure EV & Appendix Source Data [file 44319_2025_428_MOESM7_ESM.zip › Source Data for Expanded View and Appendix/FigureEV4/B/SDS_PAGE_raw_data.tif]
