# Supplementary material for: Structural insights into the selective recognition of RF-amide peptides by neuropeptide FF receptor 2
Source: EMBO Rep. 2025 Mar 24;26(9):2413–34. doi: 10.1038/s44319-025-00428-2 (PMC12069643; doi:10.1038/s44319-025-00428-2)
Supplement: Supplementary file 8 — Expanded View Figures [file 44319_2025_428_MOESM8_ESM.pdf]

Expanded View Figures

Figure EV1. Sample preparation and cryo-EM data analysis of the hNPSF–NPFFR2–G$_i$ complex.

(**A**, **B**) Size exclusion chromatography and SDS-PAGE profile of hNPSF–NPFFR2–G$_i$ complex used in the cryo-EM sample preparation. (**C**) Representative image of cryo-EM micrograph (scale bar: 140 nm) and 2D classification results. (**D**) Cryo-EM data processing workflow using CryoSPARC. (**E**) Gold standard Fourier shell correlation (GSFSC) curve and direction distribution profile. (**F**) Representative image of electron density map and model of the hNPSF–NPFFR2–G$_i$ complex. Source data are available online for this figure.

 © The Author(s)

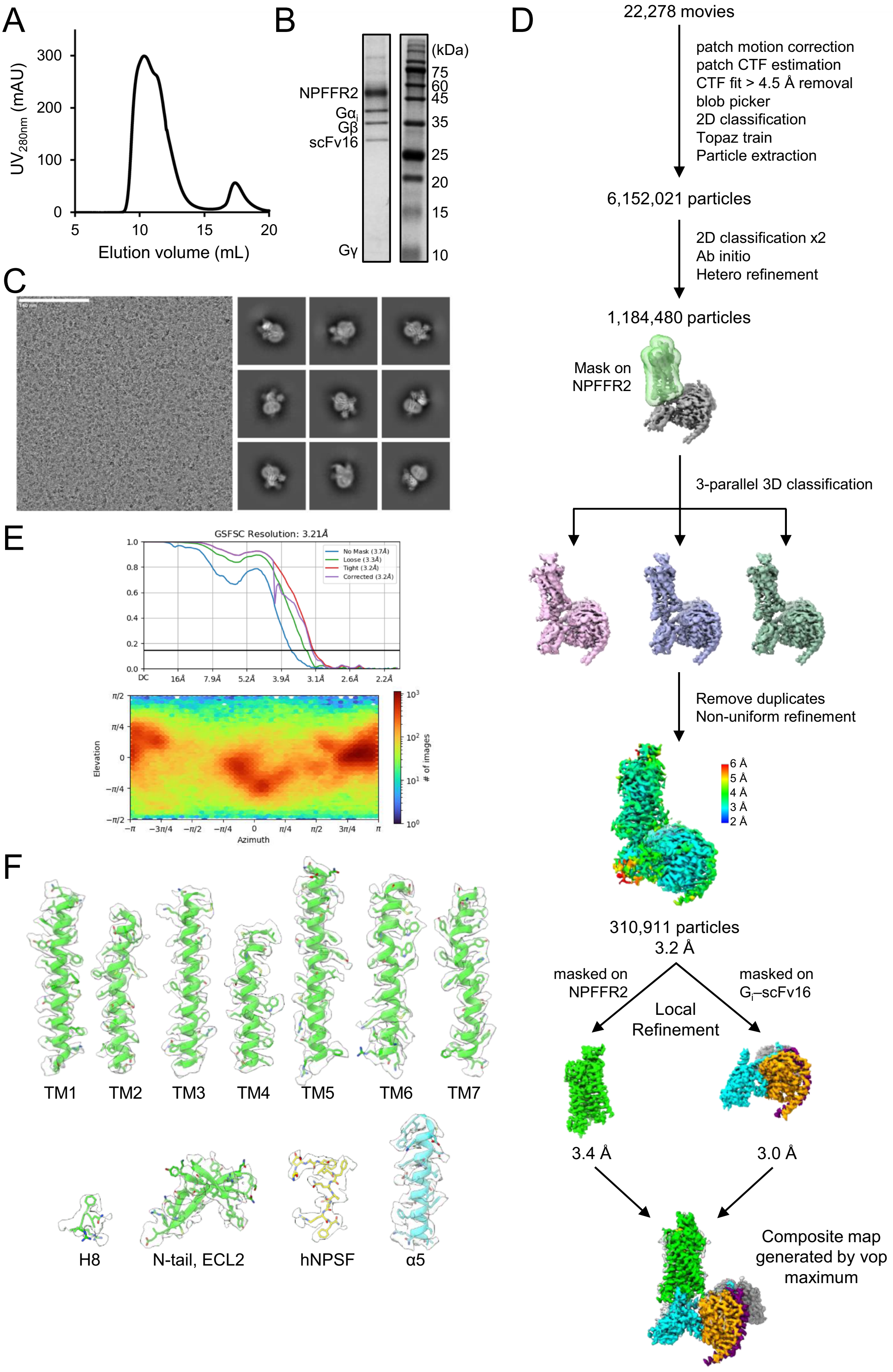
A
UV280nm (mAU)
300
200
100
0
5
10
15
20
Elution volume (mL)
B
NPFFR2
Gαi
Gβ
scFv16
Gγ
(kDa)
75
60
45
35
25
20
15
10
C
D
22,278 movies
patch motion correction
patch CTF estimation
CTF fit > 4.5 Å removal
blob picker
2D classification
Topaz train
Particle extraction
6,152,021 particles
2D classification x2
Ab initio
Hetero refinement
1,184,480 particles
Mask on NPFFR2
3-parallel 3D classification
Remove duplicates
Non-uniform refinement
6 Å
5 Å
4 Å
3 Å
2 Å
310,911 particles
3.2 Å
masked on NPFFR2
masked on Gi–scFv16
Local Refinement
3.4 Å
3.0 Å
Composite map generated by vop maximum
E
GSFSC Resolution: 3.21Å
No Mask (3.7Å)
Loose (3.3Å)
Tight (3.2Å)
Corrected (3.2Å)
DC
16Å
7.9Å
5.2Å
3.9Å
3.1Å
2.6Å
2.2Å
Elevation
Azimuth
# of images
F
TM1
TM2
TM3
TM4
TM5
TM6
TM7
H8
N-tail, ECL2
hNPSF
α5

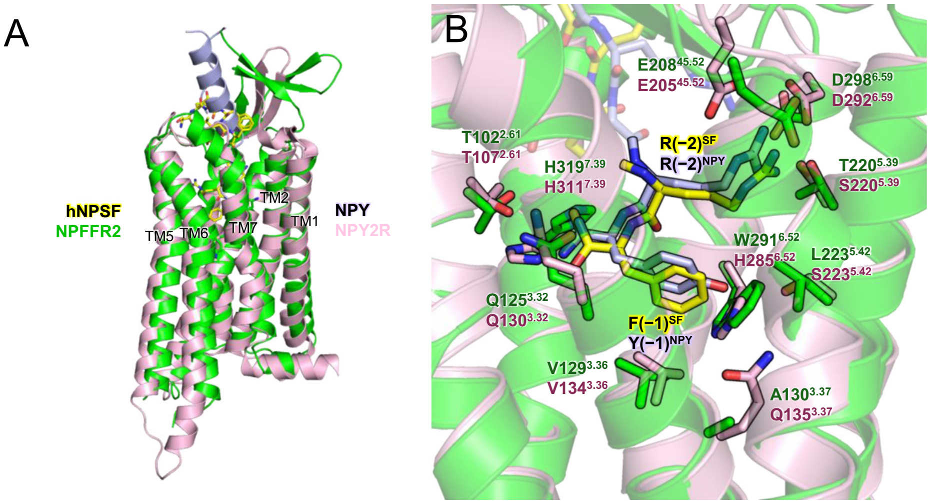

Figure EV2. Comparison of the agonist binding pockets of NPFFR2 and NPY2R.

(**A**) Superposition of the hNPSF–NPFFR2–G_i complex and the NPY–NPY2R–G_i complex (PDB: 7YOO). hNPSF is colored yellow, NPFFR2 is green, NPY is light purple, and NPY2R is pink. The structures are aligned based on the receptors, with an overall RMSD of 0.9 Å. (**B**) A detailed view at the bottom of the ligand-binding pocket of NPFFR2 and NPY2R reveals that two C-terminal residues of hNPSF and NPY share similar binding poses. NPY2R residues involved in the interaction toward Arg-Tyr-NH_2 and the corresponding residues in NPFFR2 are shown in sticks.

 © The Author(s)

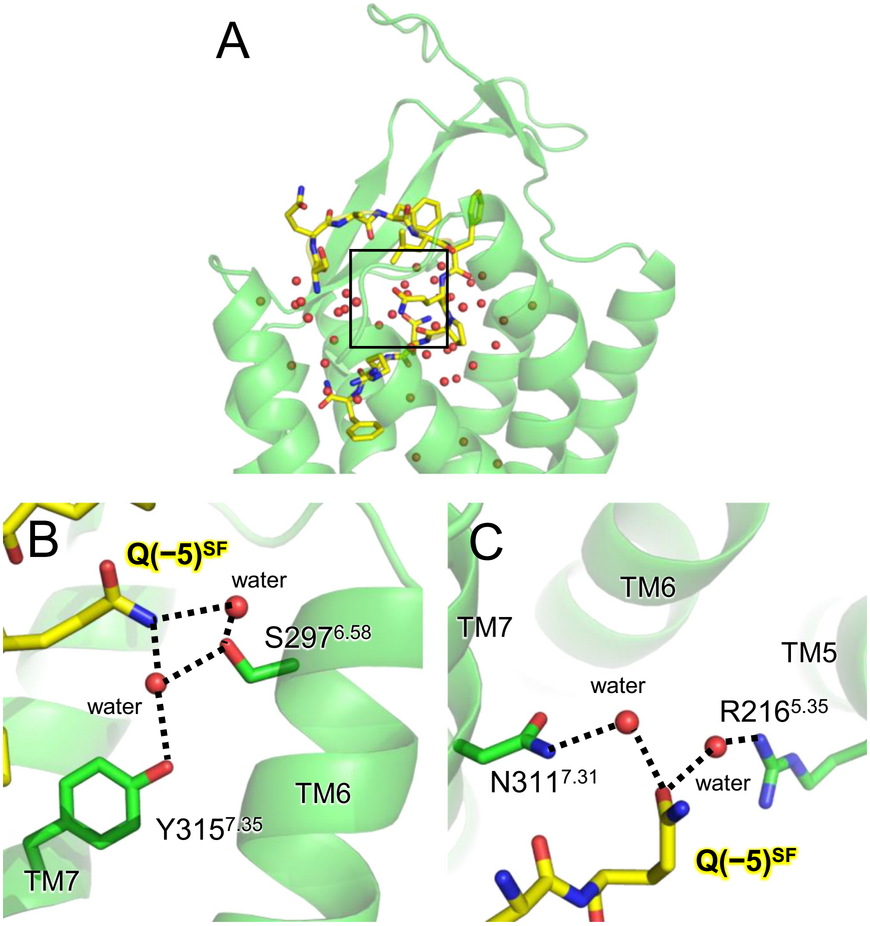

Figure EV3. Water-mediated interaction between hNPSF Q(−5) and NPFFR2.

(**A**) Water molecules near the extracellular regions of TMD are shown as red spheres in the all-atom MD simulation. (**B**, **C**) Water-mediated interactions involving Q(−5)^{SF}, based on model structures from MD simulation frames. The ligand and receptor residues (R216$^{5.35}$, S297$^{6.58}$, N311$^{7.31}$ and Y315$^{7.35}$) form hydrogen bonds with water molecules, with N and O atoms positioned within 3.5 Å.

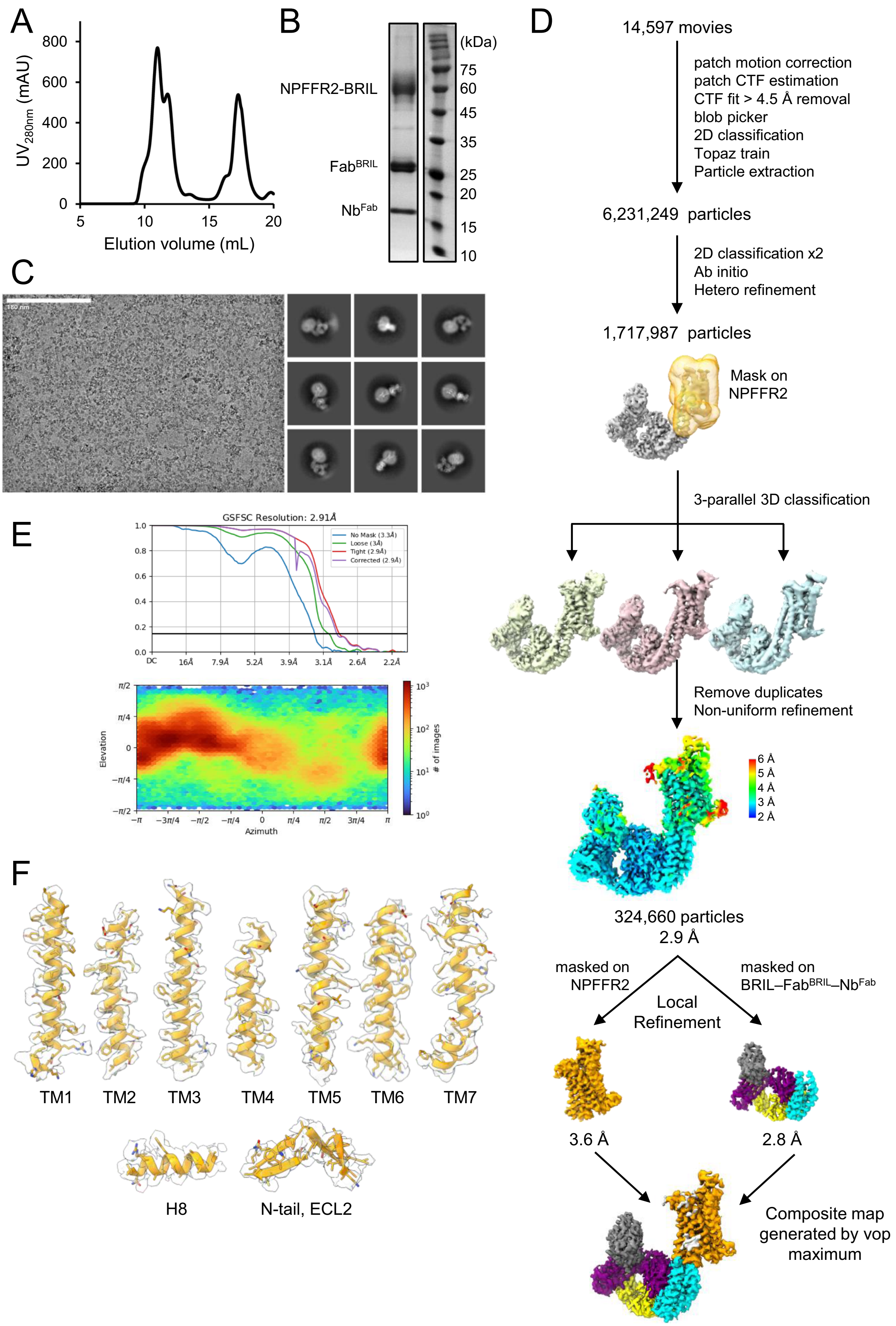
A
800
600
400
200
0
UV280nm (mAU)
5
10
15
20
Elution volume (mL)
B
NPFFR2-BRIL
FabBRIL
NbFab
(kDa)
75
60
45
35
25
20
15
10
C
D
14,597 movies
patch motion correction
patch CTF estimation
CTF fit > 4.5 Å removal
blob picker
2D classification
Topaz train
Particle extraction
6,231,249 particles
2D classification x2
Ab initio
Hetero refinement
1,717,987 particles
Mask on
NPFFR2
3-parallel 3D classification
Remove duplicates
Non-uniform refinement
6 Å
5 Å
4 Å
3 Å
2 Å
324,660 particles
2.9 Å
masked on
NPFFR2
masked on
BRIL–FabBRIL–NbFab
Local
Refinement
3.6 Å
2.8 Å
Composite map
generated by vop
maximum
E
GSFSC Resolution: 2.91Å
No Mask (3.3Å)
Loose (3Å)
Tight (2.9Å)
Corrected (2.9Å)
Elevation
Azimuth
# of images
F
TM1
TM2
TM3
TM4
TM5
TM6
TM7
H8
N-tail, ECL2

© The Author(s)

Figure EV4. Sample preparation and cryo-EM data analysis of the NPFFR2-BRIL–Fab^{BRIL} –Nb^{Fab} complex.

(**A**, **B**) Size exclusion chromatography and SDS-PAGE profile of the NPFFR2-BRIL–Fab^{BRIL}–Nb^{Fab} complex used in the cryo-EM sample preparation. (**C**) Representative image of cryo-EM micrograph (scale bar: 180 nm) and 2D classification results. (**D**) Cryo-EM data processing workflow using CryoSPARC. (**E**) Gold standard Fourier shell correlation (GSFSC) curve and direction distribution profile. (**F**) Representative image of electron density map and model of the NPFFR2-BRIL–Fab^{BRIL}–Nb^{Fab} complex. Source data are available online for this figure.

© The Author(s)
